# Development and characterization of pramipexole dihydrochloride buccal films for Parkinson’s disease treatment

**DOI:** 10.1371/journal.pone.0329142

**Published:** 2025-07-31

**Authors:** Krisztián Pamlényi, Bárbara Ferreira, Géza Regdon Jr., Katalin Kristó, Bruno Sarmento

**Affiliations:** 1 Institute of Pharmaceutical Technology and Regulatory Affairs, University of Szeged, Szeged, Hungary; 2 i3S, Instituto de Investigação e Inovação em Saúde, Universidade do Porto, Porto, Portugal; 3 INEB – Instituto de Engenharia Biomédica, Universidade do Porto, Porto, Portugal; 4 ICBAS – Instituto de Ciências Biomédicas Abel Salazar, Universidade do Porto, Porto, Portugal; 5 IUCS-CESPU – Instituto Universitário de Ciências da Saúde, Gandra, Portugal; Egaz Moniz School of Health and Science, PORTUGAL

## Abstract

Parkinson’s disease is one of the most common neurodegenerative diseases worldwide. Swallowing difficulties present a significant challenge in the treatment of Parkinson’s disease patients. Our current experimental work aimed to produce and assess a buccal polymer film containing pramipexole as the active pharmaceutical agent. This dosage form addresses the swallowing difficulties faced by Parkinson’s patients, potentially eliminating inappropriate drug application and thereby improving therapeutic success. For the preparation of polymer films, chitosan was used as the film-forming agent, and pramipexole dihydrochloride was the active pharmaceutical ingredient. The physical properties of the prepared polymer films, such as thickness, tensile strength, mass, and disintegration time were examined, along with *in vitro* mucoadhesivity. The chemical interactions between the different components of the films were analyzed using FTIR spectroscopy. Additionally, the dissolution of pramipexole from the polymer film and the permeation of the active ingredient through the TR146 buccal cell line were investigated. Finally, the biocompatibility of the prepared polymer films was also tested on a buccal cell line. The results indicate that increasing the concentration of glycerol reduced the tensile strength and mucoadhesion of the films to 12 kPa and 7 N, respectively. Interactions between the components of films were confirmed by FTIR analysis. All formulations demonstrated biocompatibility higher than 80%. Based on different investigations, Sample 4 and Sample 5 are suitable for buccal application. These formulations exhibit promising attributes for treating Parkinson’s disease.

## 1. Introduction

In recent years, the scientific community has recognized the importance of involving patients in drug development, leading to a shift towards patient centricity [1]. In the pharmaceutical technology field, this shift often involves re-formulating orally administered tablets into buccal drug delivery systems for patients with dysphagia [2]. Dysphagia, defined as an abnormal swallowing process, is a prevalent symptom in Parkinson’s disease (PD) patients [3]. The causes of dysphagia are varied and can include direct trauma, pneumothorax, weakness of oral musculature, central nervous system diseases, or side effects of drugs [4–6].

PD affects over 10 million people worldwide [7]. Oral tablets are the most commonly used form of medication therapy [8,9]. However, for patients suffering from dysphagia, swallowing tablets can be very difficult and uncomfortable. The buccal mucosa offers an alternative drug administration route that can circumvent the challenges of oral treatment [10,11]. Buccal dosage forms include tablets, films, and gels providing several benefits beyond avoiding swallowing difficulties [12,13]. Buccal medications can deliver a fast effect due to the dense vascularization of the buccal mucosa [14,15]. Additionally, buccal administration can achieve a local effect, which is useful for maintaining proper oral hygiene and treating conditions such as sore throat [16].

Moreover, the first-pass effect of the liver can be avoided with buccal administration, ensuring that the full amount of the permeated active pharmaceutical ingredient (API) is effective [17]. Buccal medications also allow for the use of acid-sensitive APIs, protecting the stomach from damage caused by the API [18,19]. In geriatrics and pediatrics, buccal drug delivery can play a significant role due to its easy and unnoticed application. This ease of use makes buccal dosage forms generally preferred by patients over oral tablets [20,21].

However, buccal dosage forms also present certain challenges. The small surface area of the buccal mucosa limits the amount of API that can be effectively absorbed, typically to doses of less than 50 mg. [22]. Additionally, buccal tablets or films should have adequate mucoadhesion properties to remain on the surface of buccal mucosa for the desired period of time. Another difficulty is that buccal films are not yet recognized as an official dosage form in Pharmacopeias and standardized studies or guidelines for their evaluation are lacking [23].

In our previous work, we formulated films containing pramipexole (PR) using sodium alginate as the polymer film forming agent [24]. Our main aim was to improve PD therapy with buccal films. The present project focuses on formulating buccal films for PD with PR and chitosan (CHI). CHI is a natural, biodegradable, cationic polymer that can bind more strongly to the buccal mucosa due to the negatively charged surface, potentially enhancing therapy [25]. Additionally, we investigated the physical properties of the films, which influence their application, storage conditions, and stability. We also investigate the chemical interactions between the components to identify any decomposition of the API or formation of new chemical agents. Furthermore, our work novelty includes the study of the biological properties of the films through cytotoxicity and permeability assays on the TR146 buccal cell line. Our ultimate goal was to find the optimal composition for buccal application, ensuring excellent mechanical properties, low cytotoxicity, and high permeability.

## 2. Materials and methods

### 2.1. Materials

CHI (low molecular weight, 130-160kDa) (Carl Roth GmbH + Co. KG, Karlsruhe, Germany) was used as a film-forming polymer. Acetic acid (AA) (min. 99.8%, Sigma-Aldrich, Darmstadt, Germany) provided the appropriate acidic environment for dissolution during the preparation of the films. Glycerol (GLY) (85% w/w) was used as a plasticizer (Ph. Eur.9.). PR (Ph. Eur. 8.) was the API, which was a gift from Krka, d.d., (Novo Mesto, Slovenia). Distilled water was used in the compositions as a dissolving medium. Mucin (Carl Roth GmbH + Co. KG, Karlsruhe, Germany) (10% w/w) dispersion was used in the in vitro mucoadhesion test. 0.05 M phosphate buffer (pH = 6.8) was used as a dissolution medium. The composition of the phosphate buffer was 1.00 g/L of KH_2_PO_4_, 2.00 g/L of K_2_HPO_4_, 8.50 g/L of NaCl.

### 2.2. Preparation of the buccal films

The buccal films were prepared at room temperature using the solvent casting method. The first step is the preparation of CHI solution. CHI was dissolved in distilled water containing AA at concentrations of 2%, 3%, and 4% (w/w). CHI is generally insoluble in water but can dissolve in acidic conditions. In this formulation, acetic acid lowers the pH of the solution, making it acidic enough to facilitate the dissolution of chitosan. Thus, acetic acid acts as a solubilizing agent, promoting the effective dispersion of chitosan in the aqueous phase and contributing to the formation of stable, uniform films. The solution was stirred continuously using a magnetic stirrer (Velp Scientifica Srl, Usmate, Italy). In the second step, PR was added to the polymer solution at a concentration of 0.0793% (w/w) for 1 h. As the third step, GLY was incorporated into the mixture at concentrations of 1, 2, and 3% (w/w). The solution was then left undisturbed for 3 hours to facilitate the reduction and removal of air bubbles. The resulting solution, with a pH range of 5.7 to 5.9 and a viscosity between 150 and 183 mPa•s, was cast onto glass surfaces in Petri dishes (6.6 cm diameter), with 10 g per dish. The films were dried at room temperature (24.4 ± 0.5 °C). Once dried, the polymer films were carefully removed from the glass surface and stored in closed containers under controlled conditions (24.4 ± 1 °C, 60 ± 2% RH) [24,26]. The different compositions of the prepared buccal films are detailed in Table 1.

**Table 1:** Composition of chitosan buccal films (composition of the polymer solution before the casting and drying process).

Samples	Chitosan % (w/w)	Acetic acid % (w/w)	GLY % (w/w)	PR (0.7 mg/cm^2^)
1	2	2	1	**+**
2	2	2	2	**+**
3	2	2	3	**+**
4	3	2	1	**+**
5	3	2	2	**+**
6	3	2	3	**+**
7	4	2	1	**+**
8	4	2	2	**+**
9	4	2	3	**+**

### 2.3. Characterization of the buccal films

#### 2.3.1. Thickness measurement.

The thickness of the polymer films was measured with a screw micrometer (Mitutoyo Co. Ltd, Japan), with a sensitivity of 0.001 mm. Measurements were taken at five randomly selected points on each film (n = 5). The mean values and standard deviations (SD) were calculated from these data [24,26,27].

#### 2.3.2. Mass determination.

To determine the mass of the polymer films, sections were cut from five different locations on each film. The mass of each 2 cm x 2 cm section was measured using an analytical balance. The mean values and SD were then calculated from these measurements [28].

#### 2.3.3. Disintegration time.

The disintegration time of the buccal films, defined as the time when a film starts to break or disintegrate, was determined by immersing 2 cm × 2 cm film samples in four milliliters of simulated saliva fluid (pH 6.8) within a glass Petri dish (diameter 9.6 cm). The dish was subjected to orbital shaking at 20 rpm at a temperature of 37 °C. The disintegration time for the fast-dissolving films was determined visually until the films were completely disintegrated and dissolved [29,30].

### 2.4. Tensile strength of films

The tensile strength of the films was investigated by a puncture test using Texture Analyser Ta-XT2i (Stable Micro Systems, Godalming, UK), equipped with a flat-faced cylindrical probe with a diameter of 7.03 mm. The software was Exponent version 6.1 which was supplied by the manufacturer. The sensitivity of the Texture Analyser 5 kg load cell is 0.001 N. Film pieces of 2 × 2 cm were fixed by screws between two plates, with a cylindrical hole with an area of 38.82 mm^2^ [30–32].

The Texture Analyser was adjusted to move the probe with a pre-test velocity of 1.0 mm/s and a gauge length of 20 mm. The test started when the probe obtained contact with the sample surface, defined by trigger force, which was set at 0.049 N. After that, the system started recording the force and displacement of the probe. The test speed was constant at 0.1 mm/s until the film ruptured. The maximum force to break (N) and distance (mm) of probe movement until break was measured, and tensile strength (N/mm^2^), Young modulus, and strain were calculated by the software [30–32].

Tensile strength was calculated using the following equation:


Tensile\ strength=force/area (1)

where the force is the measured maximum force at film rupture (N) and the area is the probe contact area with the film (mm^2^) [30–32].

### 2.5. *In vitro* mucoadhesion test of films

Mucoadhesion was tested with a self-developed device and software developed at our institute [27,33–35]. In this study, a rod-like sample holder with a diameter of 5 mm was used. A double-faced adhesive tape was fixed on the surface of the sample holder, and the polymer film was fixed to the other face of the adhesive tape. A fixed disc with a diameter of 35 mm was applied on the bottom part of the tester, and 40 µL of freshly prepared mucin dispersion (10% w/w) was spread on it. The rod-like sample holder moved to the fixed, bottom disc and pressed the polymer film to the mucin-covered bottom disc with 30 ± 0.1 N for 30 s. This steady-state part can be found in the force-time curve. Thereafter, the sample holder went back to the original place, and the force was decreased until the sample started to separate from the mucin, which can be seen as a well-defined, sharp peak in the force-time curve, indicating the *in vitro* mucoadhesion force of the films. Means and standard deviations were calculated (n = 5) [27,33–35].

### 2.6. FTIR spectroscopy

Chemical characterization of the buccal films was performed by Fourier-Transform Infrared Spectroscopy (FTIR) by using coupled Zn/Se horizontal attenuated total reflectance (HATR) equipment. The raw material and the polymer films were analyzed with a Perkin-Elmer Frontier spectrometer (Waltham, MA, USA). Films were laid on a clean crystal of the apparatus. The range of wavelength was 600–4000 cm^−1^. The spectra were collected from 64 scans, at the spectral resolution of 4 with CO_2_ and H_2_O for correction.

### 2.7. Dissolution test

Two pieces of polymer films with a size of 1 cm^2^ (containing in total 1.4 mg of PR) were investigated in the dissolution test. The dissolution test was performed by a multi-position orbital shaker (Thermo Fischer Scientific, Waltham, MA, USA) in a 100 mL glass lab beaker at a mixing speed of 100 rpm. 50 mL of 0.05 M phosphate buffer (pH = 6.8) at 37 °C was used as a dissolution medium. Aliquots of 5 mL were analyzed in 5, 10, 15, 20, 30, 40, 50, 60, 90 min with Genesys 10S UV–VIS (Thermo Fisher Scientific, Waltham, MA, USA) UV-spectrophotometry at λ = 262 nm wavelength (calibration equation: y = 0.0312x, x = concentration: µg/ml, y = absorbance; R^2^ = 0.9991). The removed volume was replaced with phosphate buffer pH = 6.8 [24,30,32].

### 2.8. Cell culture maintenance

TR146 cells were chosen to mimic the stratified epithelium of human buccal mucosa [36]. TR146 is a cell line originating from a neck node metastasis of a human buccal carcinoma. TR146 (human buccal epithelium cell line culture) cells were cultured separately in 75 cm^2^ T-flasks in Dulbecco’s Modified Eagle’s Medium (DMEM) supplemented with (Fetal Bovine Serum) FBS (10% (v/v)), NEAA (1% (v/v)) and antibiotic/antimycotic (1% (v/v) mixture (penicillin (100 U/mL) and streptomycin (100 U/mL)), inside an incubator with a humidified atmosphere at 37 °C and 5% CO_2_. Usually, the medium was changed three times per week. When confluence reached about 70% to 80%, cells were detached with trypsin-EDTA at 37 °C and seeded in another 75 cm^2^ T-flask [37].

### 2.9. Cytotoxicity assay

Briefly, buccal films were immersed in DMEM with different free drug concentrations (1, 3, and 6 µM) and incubated for up to 24 h at 37 °C, at a ratio of 2 mL medium/cm^2^ film. The samples obtained were used either undiluted (100%) or diluted with medium to 50% and 10% (v/v). The cytotoxicity of the samples was determined using the resazurin assay [37]. Cells were seeded in 96-well plates at 5000 cells per well and left under standard conditions for 24 h. Then, the samples were incubated for an additional 24 h. Cells were then washed twice with phosphate-buffered saline (PBS) (pH 7.4) before being incubated for 2 h at 37 °C in the dark with 10 μg/mL resazurin. After, fluorescence was measured at the excitation and emission wavelengths of 530 and 590 nm, respectively, using a Synergy Mx MultiMode microplate reader (BioTek, USA). Experiments were performed in triplicate, and all data were normalized regarding the positive control (medium), which was considered 100% metabolic activity [34,37].

### 2.10. *In vitro* permeability assay

TR146 cells (2 × 10^5^ cells/well) were seeded in the apical side of 6-Transwell cell culture inserts (PET, pore size 3.0 µm) [30]. The TR146 cell monolayer was kept at 37 °C and the medium changed three times weekly. The permeability assays were performed on the 24^th^ day of culture and in the apical-to-basolateral direction. The culture medium was removed from both sides of the Transwell^®^ inserts and the inserts were washed twice with pre-warmed Hank’s balanced salt solution (HBSS). After the washes, 2.5 and 1.5 mL of HBSS were placed on the basolateral and apical sides of the inserts, respectively, and they were allowed to equilibrate for 30 min at 37 °C and 100 rpm in a KS 4000 ic control orbital shaker (IKA, Staufen, Germany). After 30 min, HBSS on the apical side was replaced by the film pieces equivalent to 50 µg of pramipexole content. The free drug was used at a concentration equivalent to 50 µg per well, dissolved in HBSS. At predetermined time points (15, 30, 45, 60, 120, and 240 min), trans-epithelial electric resistance (TEER) was measured using an EVOM epithelial voltohmmeter (World Precision Instruments, Sarasota, FL, USA) to assess the monolayer integrity. A sample of 200 μL was taken from the basolateral side and replaced with the same amount of HBSS maintaining sink conditions. At the end of the assay, a sample from the apical side was taken. Moreover, the cells were subjected to lysis using 1% Triton-X to quantify the pramipexole adsorbed to the cell surface or internalized by TR146 cells [30,31]. The different samples were measured by UV/VIS spectrophotometry (Thermo Fisher Scientific, Waltham, MA, USA) at λ = 262 nm.

### 2.11. Statistical analysis

The significance tests for tensile strength and *in vitro* mucoadhesivity test were performed with Microsoft Excel (version 15, Redmond, Washington, DC, USA) software. A two-sample t-test was used. The test was done five times for each sample. To make the figures were used the GraphPad Prism software (version 8, GraphPad Software, San Diego, CA, USA). In each case, we used a significance level of *p* < 0.05. Significance is labelled as ns = *p* ≥ 0.05; * = *p* < 0.05.

## 3. Results

### 3.1. Thickness, mass, and disintegration time of the buccal films

In the context of buccal drug delivery systems, exploring thickness is crucial. If the thickness exceeds optimal levels, it may create a sensation of a foreign object, potentially hindering patient compliance. This is the reason that the buccal films have greater patient compliance than buccal tablets [2,38]. Table 2 illustrates the results of thickness measurements, indicating a correlation between CHI concentration and film thickness. Sample 1, Sample 4, and Sample 7 exhibited increased thickness with higher CHI concentrations because CHI can increase the dry matter content of the films. At the same time, this can create a stable and cohesive structure due to the larger number of polymer chains, so overall CHI can increase the thickness of the films. Additionally, the GLY can also contribute to thicker films (evident in Samples 1, Sample 2, Sample 3, etc.). This effect can be attributed to the GLY hygroscopicity and water retention effect, resulting in higher water content in films containing 2 and 3% GLY [26,39]. Wang at al. described that GLY destroys and reorganizes the polymer chain network because plasticizers can destroy the intermolecular polymer chain network and produce more free volume in the system. Therefore, incorporating a plasticizer increased the thickness of the polymer films [40]. Notably, in our films the GLY concentration exerts a greater influence on film thickness compared to the CHI concentration.

**Table 2:** Physical properties of the films (thickness, mass, and disintegration time).

Samples	Thickness (µm ± SD)	Mass of 2x2cm film (mg ± SD)	Disintegration time (min ± SD)
1	16.2 ± 3.9	30.6 ± 2.9	2.5 ± 0.4
2	73.4 ± 9.2	43.0 ± 1.6	4.6 ± 0.8
3	143 ± 10.9	64.7 ± 5.6	6.94 ± 1.2
4	33.6 ± 6.1	35.3 ± 0.7	5.66 ± 0.6
5	58.0 ± 9.2	49.3 ± 2.4	7.11 ± 1.4
6	79.2 ± 12.9	48.8 ± 3.3	9.39 ± 1.7
7	66.8 ± 12.2	62.6 ± 2.6	18.36 ± 3.5
8	78.8 ± 7.5	64.4 ± 2.6	22.20 ± 3.6
9	167 ± 14.4	71.5 ± 0.9	22.54 ± 3.4

The components of the films determine the weight of the polymer films. The mass of the films affects the successful application of the medicines, as the heavier film washed off the surface of the mucous membrane more easily and the heavier films must have greater mucoadhesion force to stay on the surface [41]. The lower concentration CHI films (Sample 1—Sample 3) have smaller weight and increasing the amount of polymer the films (Sample 7—Sample 9) have significantly higher weight due to the higher amount of dry matter as it can be followed in Table 2. The GLY concentration can also enhance the mass of the films and this effect is stronger than the increase of the CHI concentration (changes are higher between Sample 1, and Sample 2 or Sample 3 than between Sample 1 and Sample 4). Overall, CHI enhances the dry matter content of the films, while GLY’s hygroscopic properties influence both the thickness and mass of the films. The CHI films are thinner than the sodium alginate films previously formulated by our research group. However, in the case of films containing 3% GLY, CHI films are significantly thicker [24]. In terms of thickness of films the CHI films possesses better results than the sodium alginate films.

The primary condition for the medicinal effect is the disintegration of the medicinal product [42,43]. We expect rapid disintegration and dissolution in the case of buccal films because saliva can wash them out. As a result, these dosage forms cannot remain on the buccal surface for long, requiring quick disintegration and dissolution. From our formulation, the disintegration time is the shorter in case of the 2% CHI films (Sample 1, Sample 2, Sample 3) and the longest in the 4% CHI films (Sample 7, Sample 8, Sample 9) (Table 2.). The higher CHI films have a more cohesive structure because it has a higher number of chains of polymer and they can create stronger structures therefore more time is needed to break and disintegrate these films. Moreover, the stronger structure can be beneficial in terms of the tensile strength of films. The GLY also influences the disintegration time in films. As it can be seen the films with higher GLY concentration have higher disintegration time. The reason for this observation is that Samples 3, Sample 6, and Sample 9 are thicker and heavier, therefore the disintegration media needs more time to erode the films. However, the CHI concentration determines the disintegration time to a greater extent than the concentration of GLY in our formulations. Overall, 4% CHI films have a long disintegration time, making them unsuitable for use on buccal mucosa as buccal films. However, Samples 1–6 are suitable for use as buccal products based on their thickness, film mass, and disintegration time”.

### 3.2. Tensile strength measurement

**Fig 1:**
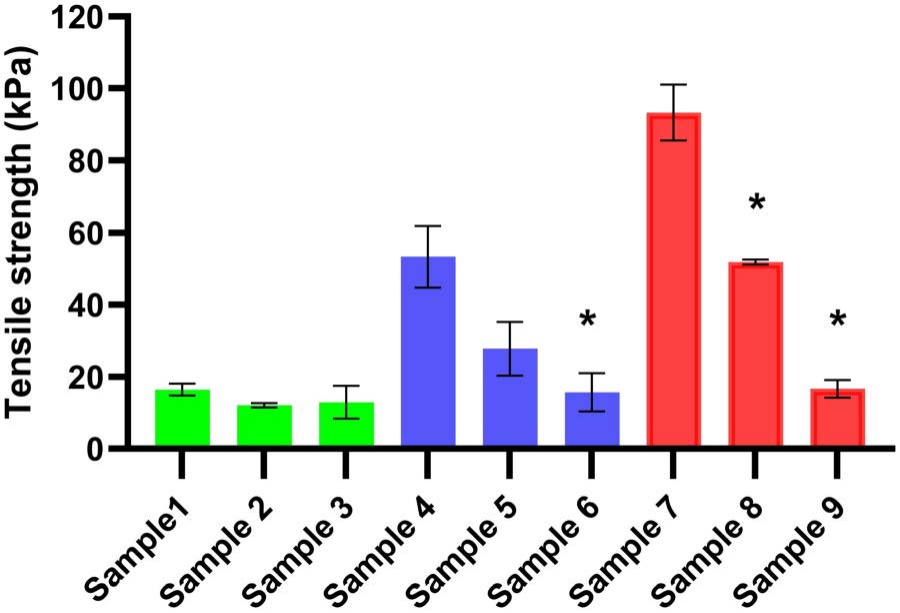
Tensile strength of the films (* p < 0.05; n = 5).

Tensile strength measurement is an important physical investigation of films that can determine their stability, storage, and application [1,2]. Films with weak tensile strength may be prone to damage, making them unsuitable for use and unable to fulfill their intended function. Fig 1 presents the results of tensile strength measurements for the formulated films. The concentrations of CHI and GLY concentrations significantly affect the tensile strength of these films. Sample 1 to Sample 3 show the lowest tensile strength values, which are the thinnest films. On the other hand, these films have the lowest CHI concentration so a cohesive and strong structure cannot be created. As the CHI concentration increases, the tensile strength of the films increases significantly. Notably, Sample 7 demonstrates a high tensile strength, exceeding 90 kPa. GLY consistently reduces the tensile strength of films in all cases. This observation can be attributed to the higher water content of the films, as previously discussed in the investigation of film mass. The high GLY concentration increases the water content, which in turn increases the bonding distance within the polymer film system. As a result, films with a high GLY concentration break more easily [26,39]. The increased water content, combined with the chemical interaction between GLY and CHI could explain this phenomenon. GLY can enhance bond distance, potentially weakening the film structure.

Overall, it can be concluded that CHI-based films exhibit significantly higher tensile strength compared to sodium alginate-based films across all GLY concentrations. This indicates that CHI is more suitable for the formulation of polymer films [24].

Young modulus can determine the flexibility of the chemical materials. As can be seen in Table 3, Young modulus can rise by the increasing polymer concentration so these films are less flexible. Young modulus is the higher in case of Sample 8 with 60.74 MPa. However, the GLY was used as a plasticizer, it can be observed that it reduces the Young modulus of samples significantly so the films are more flexible and contain a high GLY amount. Overall, the plasticizer effect of GLY was proved with numerical values.

**Table 3:** Physical properties of the films (Young modulus (MPa) and strain (mm/mm)).

Samples	Young modulus (MPa ± SD)	Strain (mm/mm ± SD)
1	24.26 ± 3.43	0.89 ± 0.07
2	9.94 ± 2.39	0.82 ± 0.13
3	14.72 ± 3.61	0.64 ± 0.19
4	47.45 ± 3.42	1.41 ± 0.19
5	33.09 ± 7.22	1.00 ± 0.22
6	12.00 ± 1.90	0.85 ± 0.11
7	49.15 ± 7.49	1.17 ± 0.17
8	60.74 ± 1.22	1.32 ± 0.12
9	19.43 ± 3.71	0.87 ± 0.08

During the measurement of the strain of the samples (Table 3), a consistent relationship between strain and material concentration was observed. Specifically, an increase in chitosan concentration led to an increase in strain, whereas glycerol (GLY) significantly decreased it. Thus, it can be concluded that films with higher Young modulus and strain values are less flexible and more resistant to pressure.

### 3.3. *In vitro* mucoadhesivity test

**Fig 2:**
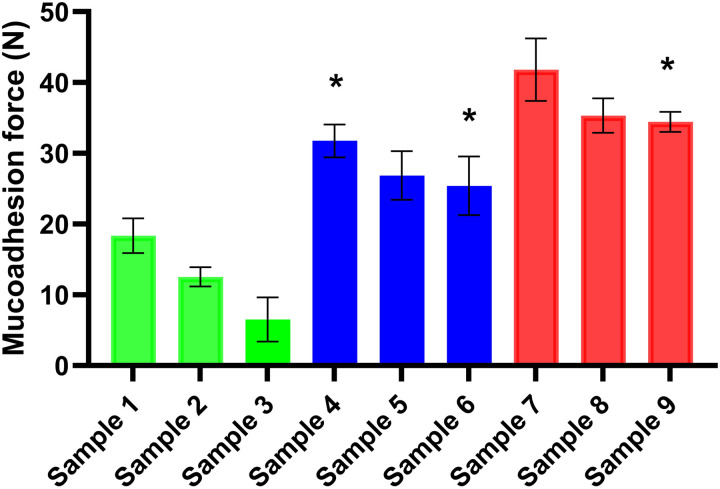
*In vitro* mucoadhesivity of the films (* p < 0.05; n = 5).

The mucoadhesivity study is a critical parameter in the development of a buccal drug delivery system. The buccal films must adhere to the surface of the buccal mucosa for 10–20 minutes to ensure the dissolution and permeation of the API [2,17]. Achieving prolonged adhesion requires high mucoadhesive force. Mucoadhesion is defined as the state in which a material and mucus or mucous membrane are held together for an extended period by attractive forces. It is defined as bioadhesion when both connecting materials are biological. This adhesion is facilitated by the interaction between the polymer chains in the film and the mucin present in the buccal mucosa, forming strong primary (covalent, ionic, ester) and secondary (hydrogen bonding, van der Waals forces) bonds with biological structures that enhance the film’s retention time [23]. Fig 2 presents the results of the *in vitro* mucoadhesion test. The 2% CHI films exhibited the lowest mucoadhesion force, whereas the 4% CHI films demonstrated the highest mucoadhesion force, around 35 N. This increased mucoadhesion in the 4% CHI films can be attributed to the higher number of polymer chains available to bind with mucin. On the other hand, GLY also influences the mucoadhesivity of the films, as illustrated in Fig 2. Increasing the GLY concentration consistently decreases the mucoadhesion force of the films. This reduction in mucoadhesion can be attributed to the chemical interaction between the chains of CHI and the hydroxyl (OH) groups of GLY, which reduces the number of CHI chains available to bind with the mucin of the buccal mucosa. Based on the literature and our previous work, the lower limit of effective mucoadhesion is determined to be 7 N, which is sufficient to ensure appropriate mucoadhesivity [26,41,44]. All films except Sample 3 exceed this limit, indicating that most formulations are suitable for buccal mucosa application. The films with 3% and 4% CHI concentrations (Sample 4 through Sample 9) show relatively strong binding to mucin.

In comparison to our previous study, where sodium alginate films were prepared with pramipexole, CHI-based films demonstrated significantly higher *in vitro* mucoadhesion forces in all cases. This is attributed to the cationic nature of CHI, which enables stronger interactions with the anionic buccal mucosa, unlike the anionic sodium alginate [24].

### 3.4. FTIR spectroscopy

**Fig 3:**
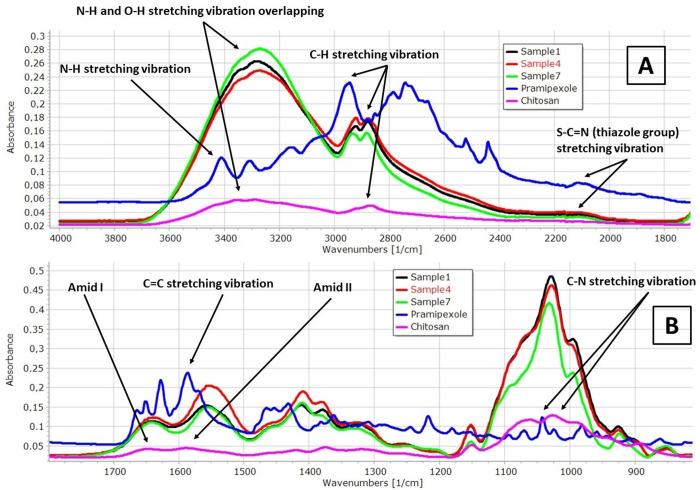
FTIR spectra of the prepared films (Sample 1: 2% CHI + 1% GLY + PR; Sample 4: 3% CHI + 1% GLY + PR; Sample 7: 4% CHI + 1% GLY + PR).

In Fig 3, the FTIR spectra of the raw materials and the different buccal films (Sample 1, Sample 4, and Sample 7) are presented. In panel A of Fig 3, the N-H stretching vibrations of the API can be observed at 3414 cm^-1^ and 3177 cm^-1^ [45]. A spectral peak at 3310 cm^-1^ is associated with the stretching vibration of the OH groups of GLY and CHI, and at 3370 cm^-1^ with the N-H stretching vibration of CHI and PR [44,46]. In the spectra of the buccal film samples, a wide peak at 3297 cm^-1^ can be observed in this wavenumber region. This peak results from the interaction between the N-H groups of CHI and PR, and the OH groups of GLY, leading to overlapping peaks and indicating the formation of intermolecular hydrogen bonds between the film components. In the lower wavenumber region, from 2922 cm^-1^ to 2873 cm^-1^, the stretching vibration of aliphatic C-H groups of PR, GLY, and CHI are visible in the spectra of the raw materials, respectively [47]. In this region of the film spectra, it can be found a double peak, suggesting that the individual stretching vibration of C-H groups overlap and interact. This interaction implies the formation of new chemical bonds between C-H groups of all components. This double peak is present in each sample.

In panel B of Fig 3, a peak at 1684 cm^-1^, in the CHI spectra corresponds to the amide I peak, while a peak at 1571 cm^-1^ corresponds to the amide II peak of CHI [48]. Additionally, the C = C stretching vibration of PR can appear at 1587 cm^-1^ [45]. The amid I and amid II peaks are observed in all film samples. However, while the amide I peak remains at the same wavelength, the amide II peak shifts towards a shorter wavelength and overlaps with the C = C stretching vibration of PR. This shift suggests the formation of intermolecular hydrogen bonds. Furthermore, the C-N stretching vibration of PR appears at 1427 cm^-1^, but this peak shifts to a lower wavenumber in the polymer film, independently of the CHI concentration, explaining an interaction between the components of the films [45].

### 3.5. Dissolution test

**Fig 4:**
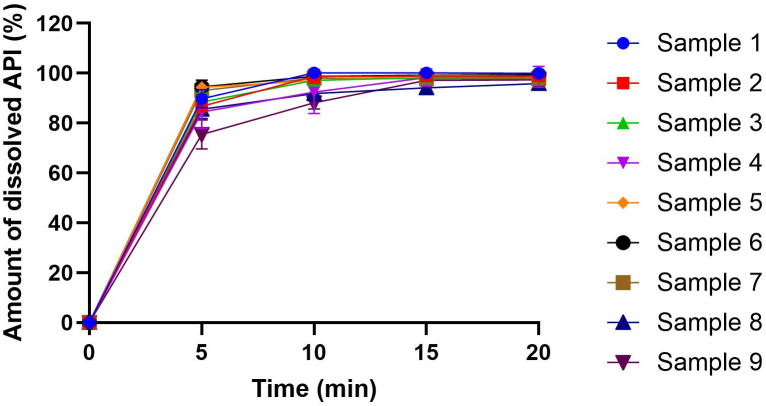
Dissolution curves of PR containing buccal films.

The dissolution study is a crucial investigation for dosage forms, especially for buccal films where fast dissolution is desired. Fig 4 presents the results of the dissolution study. In the first 5 minutes, more than 85% of PR can be dissolved from the 2% (Sample 1 to Sample 3) and 3% (Sample 4 to Sample 6) CHI films. The 4% CHI films exhibited a slower API release due to the cohesive structure formed by the higher number of polymer chains, as supported by the mass and disintegration time investigations. Within 10 minutes, more than 95% of PR was released from the 2% and 3% CHI films. While GLY did not significantly affect the dissolution of the API, the films with the highest GLY concentration showed a slightly slower release. This can be attributed to the interaction between the CHI chains and the OH groups of GLY, as observed in the FT-IR measurements. However, this difference was not significant. By 20 minutes, nearly 100% of the API was released from all film samples. Summarizing these results, it can be concluded that the 2% and 3% CHI films (Samples 1–6) are suitable for buccal application due to their rapid dissolution, also considering the disintegration times of the samples. Compared to sodium alginate films, CHI-based films released the active pharmaceutical ingredient (API) more rapidly, particularly within the first 5–10 minutes, suggesting a more favorable dissolution profile with CHI [24].

### 3.6. Cytotoxicity assay

**Fig 5:**
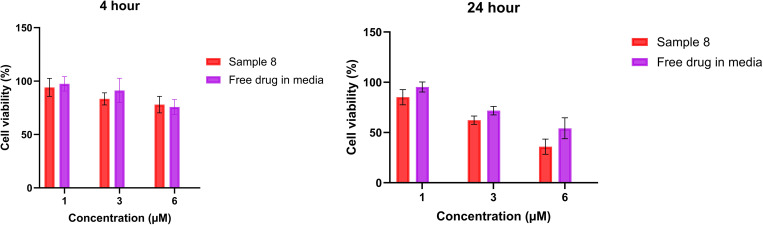
Cytotoxic effects of buccal films with 4% CHI + 2% GLY + PR (Sample 8) on TR146 cells. Results are the average of triplicates, and bars represent the standard deviation (SD).

The cytotoxicity assay is one of the standard investigations during the development of medicines. Based on different drug administration routes, it is possible to test whether a given formulation is safe or not on different cell types and different organisms. The cytotoxicity assay was performed at three different concentrations (1, 3, and 6 µM) in a shorter experiment (4 h) and a longer experiment (24 h). Fig 5 shows the cell viability results for Sample 8, which had the lowest cell viability values and was therefore chosen for presentation. In the 4-hour test, all concentrations showed high cell viability for the formulated samples, with no significant difference between the free drug and the samples. During the 24-hour test, the films were non-toxic at 1 and 3 µM concentrations but were toxic at the 6 µM concentration for TR146 cells. The longer-term experiment revealed a significant difference between the free drug and the films, as shown in Fig 5. This difference is attributed to the presence of GLY and AA in the films, which are known to be toxic based on literature [49,50]. However, buccal films can remain on the surface of buccal mucosa for a shorter time so for us, the results of the shorter-term experiment are more relevant. In summary, sodium alginate films demonstrated significantly higher cell viability values compared to the CHI films. However, based on short-term cytotoxicity results, all formulations can be considered non-toxic and suitable for buccal application..

### 3.7. *In vitro* permeability assay

**Fig 6:**
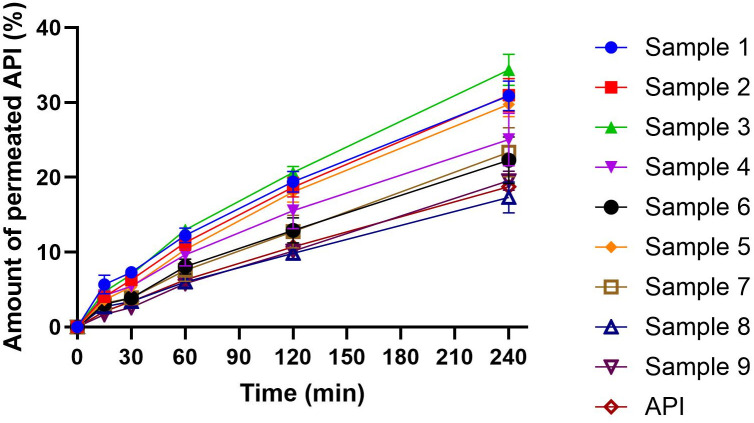
Permeability curves of the films (API reports the PR in solution with DMEM).

The permeability test is closely related to the dissolution test, however, it is a much more informative test from the point of view of the effect, since the amount of permeated API can produce an effect, while this statement is not true in the case of the dissolution test. Fig 6 presents the results of the permeability assay. The API solution, used as a reference, showed consistently lower permeation values compared to the buccal film samples. This difference can be attributed to the permeation-enhancing effect of chitosan present in the films. In all cases, the samples have a higher permeation rate on the TR146 buccal cell line due to the chitosan permeation enhancer effect [51]. Furthermore, PR permeated to a greater extent from films containing a lower concentration of CHI (2%). This can be attributed to the faster release of the active substance from these films due to the inability to form a cohesive structure at lower CHI concentrations. With 4% CHI concentration, the permeation speed and rate of API were slower and smaller. The GLY cannot influence significantly the permeation of API in the CHI films. Overall, our results align with existing literature, where permeation values of 10% or lower are typical for TR146 cells, as observed in our previous study using sodium alginate films ed [24]. In contrast, the current study achieved permeation values exceeding 30% in the best-performing formulations, and even the least effective films exhibited values above 15%. These results significantly surpass those commonly reported in the literature, indicating that our formulations have achieved excellent permeability performance [52,53]. These findings indicate that our formulations have achieved acceptable permeability outcomes.

## 4. Conclusions

The buccal drug delivery systems offers several beneficial properties. Our current research focuses on the development of buccal polymer films for the treatment of PD. Buccal films can effectively alleviate the swallowing difficulties associated with PD, thereby enhancing therapy success. In our study, we formulated films containing PR and extensively evaluated their physical, and chemical properties, as well as performed essential pharmaceutical tests relevant to buccal films.

In terms of physical tests, the thickness and weight of the films are directly affected by the concentrations of CHI and GLY. Increasing these concentrations results in thicker and heavier films. Similarly, disintegration time correlates with CHI and GLY concentrations, with higher concentrations requiring more time to disintegrate. Tensile strength tests revealed that CHI enhances film strength, whereas higher concentrations of GLY reduce tensile strength due to increased bonding distances between film materials. These effects were found in the *in vitro* mucoadhesion tests, where most samples demonstrate adequate adhesion properties for oral mucosa use. Chemically, interactions were observed between CHI, GLY, and PR, primarily in the form of hydrogen bonds. These interactions influence the speed and permeation rate of API from the films. Spectral analysis showed no significant chemical change or decomposition, confirming the stability of the films. API release was fastest from films with lower CHI concentrations, with more than 80% of API dissolved within 5 minutes and complete release within 20 minutes across all samples. This rapid dissolution meets dosage form requirements. Short-term cytotoxicity assays demonstrated that all samples were non-toxic for TR146 cells. Additionally, a 24-hour test indicated that the films were non-toxic at concentrations of 1 and 3 µM, but exhibited toxicity at a concentration of 6 µM. Permeability studies indicated that the best compositions achieved API permeation rates exceeding 30% after 240 minutes, with consistent permeation rates after 5 minutes. The increasing CHI concentration can decrease the speed and amount of the permeated API.

In conclusion, based on their physical properties, dissolution characteristics, and permeation rates, Sample 4 and Sample 5 are suitable for buccal application. These formulations exhibit promising attributes for treating Parkinson’s disease, offering fast dissolution and high API permeation rates but further *in vitro* and *in vivo* studies are needed for the final statement.

## Supporting information

S1 File S1 Tables. Dissolution data sheets. S2 Tables. Permeation data sheets.
(DOCX)

## Abbreviations

PD: Parkinson’s disease
PR: Pramipexole dihydrochloride
CHI: Chitosan
GLY: Glycerol
AA: Acetic acid
API: active pharmaceutical ingredient
FTIR: Fourier-Transform Infrared Spectroscopy
HATR: horizontal attenuated total reflectance
SD: standard deviation
PBS: phosphate buffered saline
HBSS: Hank’s balanced salt solution
DMEM: Dulbecco’s Modified Eagle’s Medium
Pen-Strep: penicillin and streptomycin
FBS: Fetal Bovine Serum
AC: apical compartment
BC: basolateral compartment
