# Supplementary material for: Development and characterization of pramipexole dihydrochloride buccal films for Parkinson’s disease treatment
Source: PLoS One. 2025 Jul 31;20(7):e0329142. doi: 10.1371/journal.pone.0329142 (PMC12312903; doi:10.1371/journal.pone.0329142)
Supplement: S1 File — S1 Tables. Dissolution data sheets. S2 Tables. Permeation data sheets. (DOCX) [file pone.0329142.s001.docx]

**Supplementary materials**

**S1 Tables Dissolution data sheets**

Dissolution data sheet – Sample1

| Time | 1st measurement (%) | 2nd measurement (%) | 3rd measurement (%) | Average (%) | SD  (%) |
| --- | --- | --- | --- | --- | --- |
| 0 | 0 | 0 | 0 | 0 | 0 |
| 5 | 88.01 | 89.51 | 91.84 | 89.78 | 2.14 |
| 10 | 97.58 | 102.49 | 100.23 | 100.10 | 1.99 |
| 150 | 97.60 | 102.51 | 100.28 | 100.12 | 1.52 |
| 20 | 99.90 | 100,51 | 98.45 | 99.92 | 1.33 |

Dissolution data sheet – Sample2

| Time | 1st measurement (%) | 2nd measurement (%) | 3rd measurement (%) | Average (%) | SD  (%) |
| --- | --- | --- | --- | --- | --- |
| 0 | 0 | 0 | 0 | 0 | 0 |
| 5 | 87.28 | 86.18 | 86.62 | 86.73 | 0.77 |
| 10 | 100.72 | 96.69 | 97.12 | 98.70 | 2.84 |
| 15 | 99.35 | 98.30 | 98.45 | 98.83 | 0.74 |
| 20 | 99.34 | 98.42 | 98.21 | 98.88 | 0.65 |

Dissolution data sheet – Sample3

| Time | 1st measurement (%) | 2nd measurement (%) | 3rd measurement (%) | Average (%) | SD  (%) |
| --- | --- | --- | --- | --- | --- |
| 0 | 0 | 0 | 0 | 0 | 0 |
| 5 | 90.93 | 85.96 | 86.73 | 88.45 | 3.52 |
| 10 | 96.46 | 97.93 | 97.13 | 97.19 | 1.04 |
| 15 | 98.75 | 97.73 | 97.25 | 98.24 | 0.73 |
| 20 | 99.48 | 97.70 | 96.95 | 98.59 | 1.25 |

Dissolution data sheet – Sample4

| Time | 1st measurement (%) | 2nd measurement (%) | 3rd measurement (%) | Average (%) | SD  (%) |
| --- | --- | --- | --- | --- | --- |
| 0 | 0 | 0 | 0 | 0 | 0 |
| 5 | 79.77 | 89.16 | 87.13 | 84.47 | 6.64 |
| 10 | 82.39 | 94.51 | 84.52 | 88.45 | 8.56 |
| 15 | 100.81 | 95.78 | 96.14 | 98.29 | 3.56 |
| 20 | 101.58 | 95.62 | 99.26 | 98.60 | 4.21 |

Dissolution data sheet – Sample5

| Time | 1st measurement (%) | 2nd measurement (%) | 3rd measurement (%) | Average (%) | SD  (%) |
| --- | --- | --- | --- | --- | --- |
| 0 | 0 | 0 | 0 | 0 | 0 |
| 5 | 95,92 | 92,78 | 93.81 | 94.35 | 2.22 |
| 10 | 98,35 | 95,68 | 98.15 | 97.02 | 1.88 |
| 15 | 98,33 | 99,90 | 99.09 | 99.11 | 1.11 |
| 20 | 98,57 | 99,18 | 99.11 | 98.88 | 0.43 |

Dissolution data sheet – Sample6

| Time | 1st measurement (%) | 2nd measurement (%) | 3rd measurement (%) | Average (%) | SD  (%) |
| --- | --- | --- | --- | --- | --- |
| 0 | 0 | 0 | 0 | 0 | 0 |
| 5 | 92.64 | 96.51 | 93.17 | 94.58 | 2.74 |
| 10 | 98.56 | 98.93 | 98.61 | 98.75 | 0.26 |
| 150 | 98.96 | 99.45 | 99.21 | 99.20 | 0.35 |
| 20 | 99.52 | 99.31 | 99.47 | 99.41 | 0.14 |

Dissolution data sheet – Sample7

| Time | 1st measurement (%) | 2nd measurement (%) | 3rd measurement (%) | Average (%) | SD  (%) |
| --- | --- | --- | --- | --- | --- |
| 0 | 0 | 0 | 0 | 0 | 0 |
| 5 | 94.87 | 91.15 | 91.41 | 93.01 | 2.63 |
| 10 | 99.55 | 96.48 | 97.17 | 98.02 | 2.17 |
| 150 | 99.40 | 96.30 | 97.86 | 97.85 | 2.19 |
| 20 | 99.09 | 96.62 | 96.70 | 97.85 | 1.74 |

Dissolution data sheet – Sample8

| Time | 1st measurement (%) | 2nd measurement (%) | 3rd measurement (%) | Average (%) | SD  (%) |
| --- | --- | --- | --- | --- | --- |
| 0 | 0 | 0 | 0 | 0 | 0 |
| 5 | 82.86 | 88.16 | 85.03 | 85.51 | 3.75 |
| 10 | 89.73 | 93.91 | 90.32 | 91.82 | 2.96 |
| 150 | 94.03 | 94.32 | 94.15 | 94.17 | 0.21 |
| 20 | 97.18 | 94.48 | 95.63 | 95.83 | 1.91 |

Dissolution data sheet – Sample9

| Time | 1st measurement (%) | 2nd measurement (%) | 3rd measurement (%) | Average (%) | SD  (%) |
| --- | --- | --- | --- | --- | --- |
| 0 | 0 | 0 | 0 | 0 | 0 |
| 5 | 79.58 | 71.39 | 76.16 | 75.48 | 5.79 |
| 10 | 89.92 | 86.31 | 88.19 | 88.11 | 2.55 |
| 150 | 97.99 | 96.25 | 96.86 | 97.12 | 1.23 |
| 20 | 98.19 | 96.55 | 97.12 | 97.37 | 1.16 |

**S2 Tables Permeation data sheets**

Permeation data sheet – Sample1

| Time | 1st measurement (%) | 2nd measurement (%) | 3rd measurement (%) | Average (%) | SD  (%) |
| --- | --- | --- | --- | --- | --- |
| 0 | 0 | 0 | 0 | 0 | 0 |
| 15 | 6.87 | 4.39 | 5.83 | 5.70 | 1.25 |
| 30 | 7.29 | 6.75 | 7.91 | 7.32 | 0.58 |
| 60 | 12.04 | 11.38 | 13.30 | 12.24 | 0.97 |
| 120 | 19.21 | 18.17 | 20.86 | 19.41 | 1.36 |
| 240 | 30.16 | 29.36 | 33.13 | 30.89 | 1.98 |

Permeation data sheet – Sample2

| Time | 1st measurement (%) | 2nd measurement (%) | 3rd measurement (%) | Average (%) | SD  (%) |
| --- | --- | --- | --- | --- | --- |
| 0 | 0 | 0 | 0 | 0 | 0 |
| 15 | 3.66 | 4.30 | 4.15 | 4.04 | 0.33 |
| 30 | 5.86 | 6.79 | 6.31 | 6.32 | 0.46 |
| 60 | 10.36 | 12.35 | 11.16 | 11.29 | 1.00 |
| 120 | 17.62 | 20.17 | 18.28 | 18.69 | 1.32 |
| 240 | 29.02 | 33.37 | 30.58 | 30.99 | 2.21 |

Permeation data sheet – Sample3

| Time | 1st measurement (%) | 2nd measurement (%) | 3rd measurement (%) | Average (%) | SD  (%) |
| --- | --- | --- | --- | --- | --- |
| 0 | 0 | 0 | 0 | 0 | 0 |
| 15 | 5.04 | 4.66 | 4.45 | 4.71 | 0.29 |
| 30 | 7.43 | 6.57 | 7.01 | 7.00 | 0.43 |
| 60 | 13.50 | 12.15 | 13.29 | 12.98 | 0.73 |
| 120 | 20.26 | 20.12 | 21.61 | 20.67 | 0.82 |
| 240 | 32.31 | 34.30 | 36.44 | 34.35 | 2.06 |

Permeation data sheet – Sample4

| Time | 1st measurement (%) | 2nd measurement (%) | 3rd measurement (%) | Average (%) | SD  (%) |
| --- | --- | --- | --- | --- | --- |
| 0 | 0 | 0 | 0 | 0 | 0 |
| 15 | 4.88 | 3.59 | 4.13 | 4.20 | 0.64 |
| 30 | 6.25 | 4.93 | 5.14 | 5.44 | 0.72 |
| 60 | 11.33 | 9.04 | 8.57 | 9.65 | 1.48 |
| 120 | 18.19 | 14.96 | 13.51 | 15.56 | 2.39 |
| 240 | 29.03 | 23.59 | 22.48 | 25.04 | 3.51 |

Permeation data sheet – Sample5

| Time | 1st measurement (%) | 2nd measurement (%) | 3rd measurement (%) | Average (%) | SD  (%) |
| --- | --- | --- | --- | --- | --- |
| 0 | 0 | 0 | 0 | 0 | 0 |
| 15 | 3.52 | 14.91 | 3.72 | 3.62 | 0.14 |
| 30 | 5.17 | 17.00 | 5.61 | 5.39 | 0.31 |
| 60 | 10.64 | 21.99 | 10.06 | 10.35 | 0.41 |
| 120 | 19.03 | 29.16 | 17.09 | 18.06 | 1.36 |
| 240 | 30.93 | 41.05 | 28.59 | 29.76 | 1.66 |

Permeation data sheet – Sample6

| Time | 1st measurement (%) | 2nd measurement (%) | 3rd measurement (%) | Average (%) | SD  (%) |
| --- | --- | --- | --- | --- | --- |
| 0 | 0 | 0 | 0 | 0 | 0 |
| 15 | 3.31 | 2.75 | 3.07 | 3.04 | 0.28 |
| 30 | 4.20 | 3.78 | 3.71 | 3.90 | 0.26 |
| 60 | 8.72 | 8.60 | 6.92 | 8.08 | 1.01 |
| 120 | 14.35 | 13.32 | 11.08 | 12.92 | 1.67 |
| 240 | 24.16 | 24.01 | 18.69 | 22.28 | 3.12 |

Permeation data sheet – Sample7

| Time | 1st measurement (%) | 2nd measurement (%) | 3rd measurement (%) | Average (%) | SD  (%) |
| --- | --- | --- | --- | --- | --- |
| 0 | 0 | 0 | 0 | 0 | 0 |
| 15 | 3.14 | 2.96 | 3.44 | 3.18 | 0.24 |
| 30 | 4.54 | 3.45 | 3.81 | 3.93 | 0.56 |
| 60 | 9.23 | 6.39 | 7.10 | 7.57 | 1.48 |
| 120 | 15.02 | 10.65 | 12.54 | 12.73 | 2.19 |
| 240 | 26.99 | 20.71 | 22.31 | 23.34 | 3.26 |

Permeation data sheet – Sample8

| Time | 1st measurement (%) | 2nd measurement (%) | 3rd measurement (%) | Average (%) | SD  (%) |
| --- | --- | --- | --- | --- | --- |
| 0 | 0 | 0 | 0 | 0 | 0 |
| 15 | 2.68 | 2.38 | 3.05 | 2.70 | 0.48 |
| 30 | 3,41. | 3.04 | 3.80 | 3.42 | 0.54 |
| 60 | 5.82 | 5.79 | 6.22 | 6.01 | 0.30 |
| 120 | 10.02 | 10.16 | 9.57 | 9.86 | 0.41 |
| 240 | 16.10 | 18.77 | 15.84 | 17.31 | 2.07 |

Permeation data sheet – Sample9

| Time | 1st measurement (%) | 2nd measurement (%) | 3rd measurement (%) | Average (%) | SD  (%) |
| --- | --- | --- | --- | --- | --- |
| 0 | 0 | 0 | 0 | 0 | 0 |
| 15 | 1.56 | 1.83 | 1.64 | 1.68 | 0.14 |
| 30 | 2.64 | 2.72 | 2.49 | 2.62 | 0.12 |
| 60 | 5.95 | 6.10 | 5.40 | 5.82 | 0.37 |
| 120 | 10.61 | 10.35 | 9.52 | 10.16 | 0.57 |
| 240 | 20.01 | 20.52 | 18.13 | 19.55 | 1.26 |
